# Multiphysics Phase-Field Modeling of Corrosion-Induced Degradation in Unsaturated Reinforced Concrete Structure

**DOI:** 10.3390/ma18225091

**Published:** 2025-11-09

**Authors:** Aihua Lu, Yongxing Zhang

**Affiliations:** 1 School of Civil Engineering, Nanjing Forestry University, Nanjing 210037, China; 2 School of Civil Engineering/Jiangsu Provincial Highway Intelligent Detection and Low-Carbon Maintenance Engineering Research Center, Nanjing Forestry University, Nanjing 210037, China

**Keywords:** unsaturated RC structure, degradation, concrete cracking, rebar corrosion, chloride ingress, multi-physics field, phase field, modeling

## Abstract

Corrosion-induced cracking poses a significant threat to the longevity of reinforced concrete (RC) structures, yet precisely forecasting its advancement continues to be a considerable scientific obstacle. The principal shortcoming of current numerical models is their excessive simplification, frequently presuming totally saturated conditions and disregarding the dynamic interplay between environmental (hygro-thermal) variations and developing mesoscale damage. This study presents a thorough hygro-thermo-electro-chemo-mechanical (HTECM) phase-field model to fill this research need. The model uniquely combines dynamic unsaturated hygro-thermal transport with multi-ion reactive electrochemistry and meso-scale fracture mechanics. A rigorous comparison with published experimental data validates the model’s exceptional accuracy. The anticipated progression of fracture width exhibited remarkable concordance with experimental data, indicating a substantial enhancement in precision compared to uncoupled, saturated-state models. A key finding is the quantification of the damage-induced “transport-corrosion” positive feedback loop: initial corrosion-induced microcracks significantly expedite the transport of local moisture and corrosive agents, leading to nonlinear structural degradation. This work presents a high-fidelity numerical platform that enhances the understanding of linked deterioration in materials science and improves the durability design of reinforced concrete structures.

## 1. Introduction

**Figure 1:**
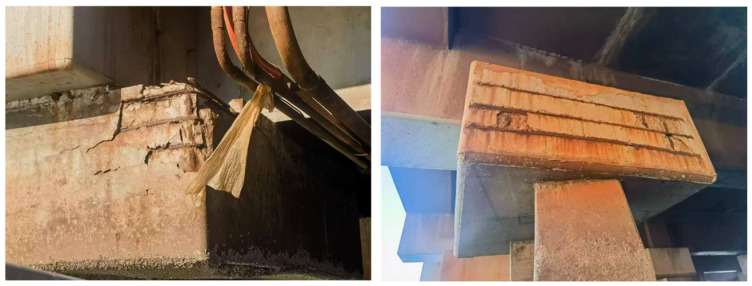
Corrosion of steel rebars in metro concrete and dock concrete.

In the mid-20th century, industrialized countries initiated a surge of infrastructure development [1]. Material aging driven by prolonged exposure to harsh environmental conditions—such as high chloride load or alternating wet-dry cycles—has become the principal concern linked to the ongoing utilization of these failing buildings [2,3,4,5]. In reinforced concrete (RC) structures often subjected to chloride-laden conditions, such as those typical in offshore infrastructure, rebar corrosion is a significant durability concern. Corrosion produced by chloride and carbonation corrosion is the most prevalent cause of corrosion-induced damage. Cracking in RC structures constitutes 70–90% of premature degradation in concrete edifices [6,7,8]. Figure 1 depicts common instances of corrosion-related deterioration in actual reinforced concrete structures. To guarantee the secure and stable functioning of RC structures, it is essential to examine the process of rust-induced expansion cracking, assess potential failure modes of the concrete cover, and evaluate the factors influencing rust expansion damage. This facilitates the forecasting of structural fracture patterns and their timing. Presently, three principal methodologies are employed to examine and forecast the longevity of RC structures: environmental corrosion test [6], artificial accelerated corrosion test [9,10], and numerical simulation [11,12,13,14,15].

The scientific community has extensively studied the intricate relationship between harsh environmental conditions and material degradation in practical service scenarios [16,17,18,19,20,21,22,23]. Researchers have examined the corrosion deterioration of concrete in wet-dry cycling conditions [16,17,18,19], with empirical findings indicating that alternating cycles create intricate moisture gradients, hence markedly expediting ion penetration and subsequent concrete cracking failure. Concurrently, other studies have focused on the degradation mechanisms of concrete under the influence of composite salts or applied loads [24,25,26,27,28,29]. To enhance comprehension of the intricate, interrelated phenomena observed in experiments and to create predictive instruments, numerous researchers have concentrated on simulating chloride corrosion under multifaceted environmental conditions, such as the alternating wetting-drying cycles prevalent in service, which are recognized to expedite ion ingress and electrochemical reactions. Nonetheless, a considerable problem in these numerical models persists: the successful integration of the intricate, dynamic environmental fields (e.g., moisture, temperature, chemical concentrations) with the onset and progression of mechanical damage.

The phase field method (PFM) has gained popularity in recent years for its capacity to combine multiphysics models in the corrosion processes of RC structures, including metal dissolving and crack nucleation and propagation. Wu [11,12,13] pioneered the unified phase-field theory and the phase-field regularized cohesive zone model (PF-CZM) in concrete fracture mechanics, studying damage mechanics and quasi-brittle fracture. Later, this paradigm was developed to solve multiphysics problems like shrinkage cracking in quasi-brittle materials like concrete. Fang et al. [30] studied electrochemical-mechanical interaction to capture non-uniform corrosion. Their model still simplifies environmental fields by failing to intrinsically couple dynamic wet-heat transport processes with electrochemical and damage evolution, which determine the spatiotemporal variation of corrosion rates in real environments. Korec et al. [31,32] improved chemical-mechanical coupling modeling by describing corrosion-driven crack development. This model series assumes concrete pores are entirely saturated, which limits its utility to simulating unsaturated conditions dominated by wet-dry cycles in engineering structures like bridges and buildings. Qiu et al. [33] used a biphasic field technique and extensive electrochemical-mechanical coupling to simulate the complete time-dependent chloride-induced corrosion and cracking mechanism with multiphysics interactions.

Most current models presume that concrete is in a perfect saturated condition, which does not correspond with the dynamic, unsaturated environments of most structures, thereby restricting the precision of forecasts. Moreover, current corrosion-cracking models mostly presume concrete to be a homogeneous substance or that particles are spherical, which is inconsistent with the true physical characteristics of concrete and the shapes of aggregates, disregarding the influence of actual aggregate geometry on the corrosion process. These deficiencies represent a significant barrier to high-fidelity, long-term durability forecasting.

## 2. Theoretical Framework and Governing Equations

Freshly poured concrete’s high alkalinity (pH 12–13) forms a deep, nanoscale passivation coating on rebar, protecting it. Outdoor elements destroy their concrete coating while working. Excessive chloride ions (Cl^−^) on rebar surfaces, especially in marine settings, can impair passivation coating and produce pitting corrosion [2]. Carbon dioxide from the environment lowers concrete pore solution pH by reacting with calcium hydroxide. Under pH 9, the passivation layer dissolves, quickening corrosion [34].

After the passivation film breaks, rebar develops microscopic electrochemical corrosion cells. Upon dissolution, rebar iron components release ferrous ions (Fe^2+^). Iron ions in concrete pore solution oxidize and hydrolyze to generate rust. Corrosion product buildup causes most concrete cracking and mechanical property degradation [35,36].

### 2.1. Overall Framework of the Model

The architecture of the fully coupled Hygro-Thermo-Electro-Chemo-Mechanical (HTECM) model is presented in two distinct flowcharts, addressing both the model’s physical phenomenology and its computational methodology.

**Figure 2:**
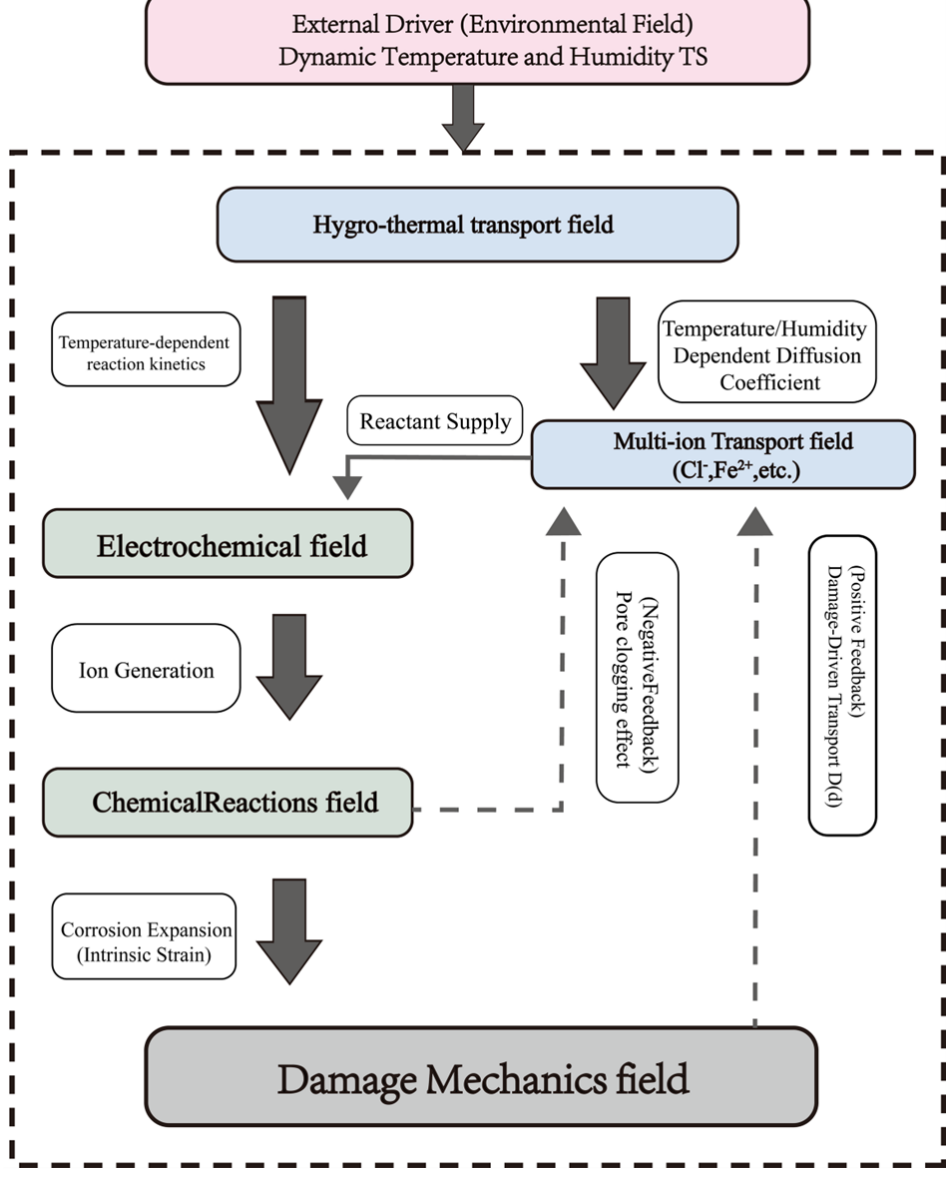
The overall framework of the proposed Hygro-Thermo-Electro-Chemical-Mechanical (HTECM) fully coupled model.

**Figure 3:**
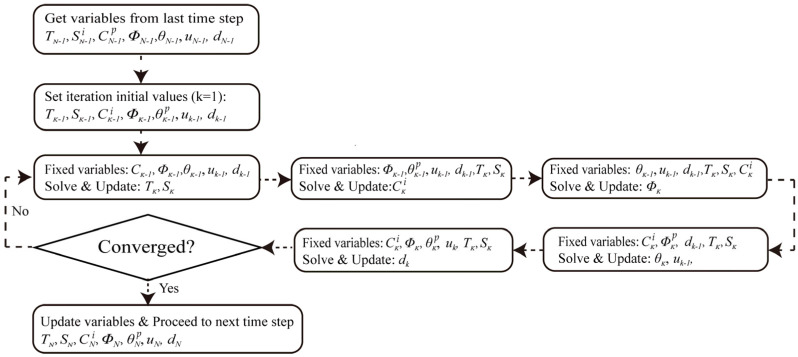
Methodological flowchart detailing the staggered (Gauss-Seidel) iterative solution scheme (logical iterations) used to solve the coupled HTECM model at each time step.

Figure 2 and Figure 3 depict the phenomenological framework of the model. It delineates the physical causal chain, commencing with exterior environmental stimuli (dynamic temperature and humidity) and progressing through the five interconnected physical domains. It also emphasizes the essential positive feedback loop (damage-driven transport) and negative feedback loop (pore-clogging effect) that regulate the deterioration. Figure 2 delineates the computing process and the exact logical iterations employed in the numerical simulation. The HTECM model is resolved with a staggered iterative methodology. At each time step *T*_*N*−1_, the system iterates (*k* = 1, 2, …). In each iteration *k*, the physical fields are resolved progressively, utilizing the values from the preceding iteration k − 1 as “fixed variables.” The process continues iteratively until convergence is achieved, at which juncture the model progresses to the subsequent time step *T_N_*.

### 2.2. Hygro-Thermal Transport in Concrete

The disintegration of concrete necessitates the transport of water. The migratory behavior in porous materials elucidates physicochemical processes such as chloride ion infiltration, carbonation, and internal corrosion expansion. Humidity, temperature, the tiny pore structure of concrete, and internal saturation influence water movement. This study effectively depicts the dynamic moisture field in concrete by employing a diffusion equation to elucidate water transport in unsaturated porous media. The primary innovation is its moisture diffusion coefficient (*D_w_*), which varies with the saturation of the concrete. This facilitates precise simulation of water’s intricate nonlinear dynamics throughout moisture absorption and desorption cycles. Equation for water flow:

(1) $$\frac{\partial s(x,t)}{\partial t}=\;\frac{\partial }{\partial x}\left(D_{w}\frac{\partial s(x,t)}{\partial x}\right),$$

The moisture hysteresis observed in concrete during moisture absorption and desorption leads to variability in its water diffusion coefficient. This coefficient, a critical parameter influencing the rate of water transport, requires distinct definitions depending on the material’s state, whether dry or wet [37,38]. The water diffusion coefficient is calculated based on variations in the saturation of the pore solution as follows [39,40]:

(2) $$D_{w}^{c}=\left\{\begin{array}{c} g(T)g(\phi )D_{w0}^{d}\frac{3{\left(1-v_{agg}\right)}^{2}}{3-v_{agg}}\left[\gamma_{0}+\frac{1-\gamma_{0}}{1+{\left(\frac{1-s}{1-s_{c}}\right)}^{n_{1}}}\right]\;\left(\mathrm{Desorption}\;\mathrm{process}\right)\\ g(T)g(\phi )D_{w0}^{s}\frac{3{\left(1-v_{agg}\right)}^{2}}{3-v_{agg}}e^{n_{2}s}\;\left(\mathrm{Adsorption}\;\mathrm{process}\right) \end{array}\right.\;,$$

$D_{w0}^{d}$ and $D_{w0}^{s}$ represent the reference water diffusion coefficients of concrete in saturated and dry limit states, respectively. Parameter $v_{\mathrm{agg}}$ denotes the volume fraction of coarse aggregate. The $\gamma_{0}$ parameter denotes the ratio of the diffusion coefficient under conditions of extremely low humidity to the saturation diffusion coefficient $D_{w0}^{d}$. $s_{c}$ represents a crucial saturation parameter. Parameters $n_{1}$ and $n_{2}$ are empirical constants that characterize the shape of the diffusion coefficient curve, governing the downward trend during drying and the upward trend during wetting. To account for temperature variations on the moisture diffusion coefficient, the subsequent formula is applied for correction [40,41]:

(3) $$g(T)=\exp[\frac{U_{h}}{R}(\frac{1}{T_{ref}}-\frac{1}{T})],$$

In this temperature influence function, $U_{h}$ represents the activation energy for the water diffusion process (35,000 J/mol), R is the gas constant, T is the absolute temperature of the current environment, and $T_{\mathrm{ref}}$ serves as the reference temperature for comparison.

The model considers the impact of pore volume fraction. This is accomplished via a function derived from the Kozeny–Carman model [42], utilizing porosity as the variable. The initial porosity $\phi_{0}$ of the cement mortar matrix is determined by the water–cement ratio (*w*/*c*) and the initial degree of hydration $\alpha_{0}$ [43]:

(4) $$q(\phi )={\left(\frac{\phi }{\phi_{0}}\right)}^{3}{\left(\frac{1-\phi_{0}}{1-\phi }\right)}^{2},\;\varphi_{L,0}=\frac{w/c-0.36\alpha_{0}}{w/c+0.32},$$

### 2.3. Multi-Ion Reactive Transport Under Coupled Environments

This study examines nine chemical species intricately associated with the corrosion process, covering the entire continuum from the ingress of corrosive ions to the generation of rust products. The substances consist of corrosive elements (Cl^−^, O_2_), ions that create the alkaline pore solution environment (Ca^2+^, OH^−^, Na^+^, K^+^), and other corrosion byproducts from rebar (Fe^2+^, Fe(OH)_2_, Fe(OH)_3_). The model incorporates three essential physicochemical mechanisms—diffusion, convection, and electromigration—alongside chemical processes to precisely depict the complex behavior of ions in porous media. The interrelated effects are depicted by a system of partial differential equations, encompassing mass conservation equations and the Nernst–Planck equation, enabling the dynamic simulation of the spatiotemporal evolution of concentration fields for each substance. The model’s governing equations are delineated as follows.

(5) $$\left\{\begin{array}{c} \frac{\partial c_{i}}{\partial t}+\nabla \cdot J_{i}=R_{i}\\ J_{i}=-D_{i}\nabla c_{i}-\frac{z_{i}F}{RT}D_{i}c_{i}\nabla \psi_{l}+c_{i}u \end{array}\right.,$$

The ion diffusion process in the equation is characterized by certain fundamental physicochemical factors. The term diffusion flux denotes the movement of each ion along a concentration gradient, with $D_{i}$ representing the ion diffusion coefficient and $c_{i}$ indicating the ion concentration. The variable $z_{i}$ denotes the charge number of the migrating ion *i*. The electrolyte potential facilitates the directional migration of ions. The Faraday constant (F), universal gas constant (R), and absolute temperature (T) collectively provide a proportionality factor that transforms electrical potential energy into chemical energy, hence elucidating the electromigration behavior of ions in response to an electric potential difference. Table 1 presents the specific diffusion coefficients and starting concentrations of various compounds.

The ion diffusion coefficient $D_{i}$ must consider variations in porosity and temperature effects. Ion diffusion is affected by the saturation level of pores in concrete; it accelerates at high saturation and decelerates at low saturation. The diffusion coefficient is articulated as follows:

(6) $$D_{i}=g(T)g(\phi )F(s)=\frac{\delta }{\tau^{2}}q(\phi )\exp\left[-\frac{E_{i}}{R}\left(\frac{1}{T}-\frac{1}{T_{ref}}\right)\right]{\left[1+\frac{{\left[1-s(t)\right]}^{4}}{{\left(1-s_{c}\right)}^{4}}\right]}^{-1}D_{i0},$$

where u represents the macroscopic velocity vector of water within the pore. This term denotes the passive transport of solute concurrent with water transport, occurring with the movement of the matrix solution. The subsequent equation delineates the convective component of ion diffusion.

(7) $$J_{i}=C_{i}u=-C_{i}D_{s}\frac{\partial s(x,t)}{\partial x},$$

Chemical reaction term $R_{i}$ describes the source or sink of each ion within the pore solution. The consideration of chloride ions in this chemical reaction is essential for accurately modeling rebar corrosion. Chloride ions, while functioning as a catalyst in the corrosion chain reaction and not being reactants on a macroscopic scale, substantially influence the effective concentration of free chloride ions in the pore solution through their interaction with the cementitious matrix. This study differentiates between mobile free chloride ions and immobilized bound chloride ions to elucidate the complexity of this mechanism. The binding rate adheres to a linear driving force model predicated on the Freundlich isotherm; hence, the diffusion equation for chloride ions is:

(8) $$\frac{\partial c_{cl}}{\partial t}+\nabla \cdot \left(-D_{cl}\nabla c_{cl}-\frac{z_{cl}F}{RT}D_{cl}c_{cl}\nabla \psi_{l}+c_{cl}\left(-C_{cl}D_{s}\frac{\partial s(x,t)}{\partial x}\right)\right)-R_{cl}=0,R_{cl}=\alpha C_{cl}^{\beta }$$

This work used a temperature-dependent Freundlich model to characterize the adsorption-binding behavior of Cl^−^ in concrete. This model more precisely represents the nonlinear fluctuations in Cl^−^ binding capacity across varying temperature settings, as opposed to a mere linear correlation. The parameters α and β are now polynomial functions of temperature *T*, defined as follows [45]:

(9) $$\left\{\begin{array}{l} \alpha =0.00002T^{2}+0.0006T+0.0775\\ \beta =0.0001T^{2}-0.0031T+0.3995 \end{array}\right.,$$

Corresponding chemical reactions leading to ion generation and reduction should be considered for other ions in the pore solution. In this model, we account for the following primary chemical reactions.

(10) $$\left\{\begin{array}{c} \begin{array}{l} 2Fe+O_{2}+2H_{2}O\to 2Fe^{2+}+4OH^{-}\\ Fe^{2+}+2OH^{-}\to Fe{\left(OH\right)}_{2} \end{array}\\ 4Fe{\left(OH\right)}_{2}+O_{2}+2H_{2}O\to 4Fe{\left(OH\right)}_{3}\\ Fe{\left(OH\right)}_{3}\to FeO(OH)\cdot H_{2}O \end{array}\right.,$$

In the governing equations of ion transport, the precise description of chemical reaction source-sink terms is critical to model fidelity. Regarding the corrosion process of iron, the pathway from the anode dissolution of ferrous ions (Fe^2+^) to the formation of the final stable rust product involves the generation of multiphase intermediate products such as ferrous hydroxide (Fe(OH)_2_), making the process highly complex. The reaction rate of the continuous transformation from ferrous ions to precipitation can be described as follows [46,47]:

(11) $$\left\{\begin{array}{l} R_{Fe^{2+}}=-k_{p_{2}}C_{Fe^{2+}}\cdot C_{OH^{-}}^{2}\\ \frac{\partial C_{p_{2}}}{\partial t}=R_{p_{2}}=k_{p_{2}}C_{Fe^{2+}}\cdot C_{OH^{-}}^{2}\\ \frac{\partial C_{p_{3}}}{\partial t}=R_{p_{3}}=-k_{p_{3}}C_{p_{2}}^{4}\cdot C_{O_{2}} \end{array}\right.,$$

Under identical pH conditions, Fe(OH)_3_ exhibits lower solubility than Fe(OH)_2_, once Fe^2+^ is oxidized to Fe^3+^, Fe(OH)_3_ precipitation forms immediately, even in relatively neutral environments. This study’s primary predictive objective is macro-cracking caused by the accumulation of corrosion products, predominantly influenced by the final product Fe(OH)_3_, which is the most voluminous and chemically stable. Consequently, we have streamlined the intermediate reaction pathways. This simplification is accomplished by establishing a high reaction kinetic rate constant, with the production rate of Fe(OH)_2_ defined as $k_{p2}\;=\;2\;\times \;10^{-4}s^{-1}$. The rate constant for the production of Fe(OH)_3_ is =0.1 $\mathrm{mo}l^{-1}m^{3}s^{-1}$. This configuration guarantees the swift oxidation of any freshly generated Fe(OH)_2_ to Fe(OH)_3_, thereby keeping the concentrations of the intermediate products Fe(OH)_2_ and Fe^2+^ at minimal levels. As a result, Fe(OH)_3_ emerges as the unequivocally predominant solid-phase product.

The model differentiates the transport behavior of different substances. For stationary solid-phase sediments, the total flow is zero; hence, the time-dependent rate of their volume fraction is exclusively governed by the chemical reaction sink term *R_i_*. Consequently, the formula for calculating solid-phase products produced by reactions is as follows:

(12) $$\frac{\partial C_{i}}{\partial t}=R_{i},\;i=p2,p3,$$

### 2.4. Electrochemistry Under Coupled Environments

Rebar corrosion fundamentally involves an electrochemical process influenced by the potential difference between the anode and cathode areas on the rebar surface. This process adheres to the laws of charge conservation and electrode kinetics, and is influenced by external factors including temperature and reactant concentration. We utilize an electrochemical framework to model this intricate process, which considers the heterogeneous nature of corrosion.

(13) $$\left\{\begin{array}{l} Fe\to Fe^{z+}+ze^{-}\\ O_{2}+2H_{2}O+4e^{-}\to 4OH^{-} \end{array}\right.,$$

The principal anode reaction in rebar corrosion involves the oxidation of iron, whereas the cathode reaction generally entails the reduction of oxygen and water, thereby preserving charge neutrality. The fundamental parameter for measuring the reaction rate is the corrosion current density, which serves as the coupling variable connecting the electrochemical and chemical transport domains. The Tafel equation is a notably reduced version of the Butler-Volmer equation under conditions of strong polarization. The general form is as follows:

(14) $$\left\{\begin{array}{l} i_{a}^{Fe}=i_{0}^{Fe}\exp\left(\frac{E-E_{eq}^{Fe}}{\beta_{a}^{Fe}\times \left(0.525\times C_{Cl^{-}}^{-1.126}+1\right)}\right)\;\\ i_{c}^{ORR}=-i_{0}^{ORR}\frac{c_{O_{2}}}{c_{O_{2,s}}}\exp\left(-\frac{E-E_{eq}^{O_{2}}}{\beta_{c}^{ORR}}\right) \end{array}\right.,$$

Anodic and cathodic current densities ($i_{a}^{\mathrm{Fe}}$ and $i_{c}^{\mathrm{ORR}}$) are related to their exchange current densities ($i_{0}^{\mathrm{Fe}}$ and $i_{0}^{\mathrm{ORR}}$), overpotentials, and equilibrium potentials ($E_{\mathrm{eq}}^{\mathrm{Fe}}$ and $E_{\mathrm{eq}}^{O_{2}}$) at the anode and cathode, respectively. Corrosion on rebar surfaces experiences a de-passivation process initiated when local chloride ion (Cl^−^) concentrations attain a critical threshold. The Tafel slope is a crucial parameter in electrode kinetics, frequently regarded as a constant in conventional models to facilitate computations. This simplification neglects the intricacy of the corrosion start phase. This model addresses this restriction by utilizing the research of Xia et al. [48]. Research characterizes the anode Tafel slope $\beta_{a}^{\mathrm{Fe}}$ as a constant function of the local chloride ion (Cl^−^) concentration. This nonlinear connection accurately mimics the gradual transition of the passivation film from stable to disturbed states, facilitating a seamless shift from passivation to activation states, which more accurately represents reality than conventional abrupt transition models. The exchange current density exhibits strong temperature dependence, and its variation can significantly alter the corrosion rate. The influence of temperature on both anode and cathode exchange current density is analyzed using the Arrhenius-type relationship, expressed by the following equation:

(15) $$i_{0}^{i}=i_{0}\exp[\frac{E_{i}}{R}(\frac{1}{T_{ref}}-\frac{1}{T})],$$

### 2.5. Damage Fracture Mechanics Under Coupled Environments

In the mechanical analysis module of this study, we have defined distinct constitutive behaviors for concrete and rebar. Rebar’s mechanical behavior is assumed to be ideal linear elastic. Concrete’s complex nonlinear fracture process is described by the phase-field cohesive zone model (PF-CZM) developed by Wu et al., which reproduces the energy dissipation and stress softening characteristics of quasi-brittle materials within the fracture process zone (FPZ)—a critical aspect for accurate simulation [11,49].

Within the chemical-mechanical coupling framework of this model, the internal stress field induced by corrosion product precipitation is introduced via the eigenstrain method. This method describes localized, stress-free deformations within materials caused by non-mechanical factors such as phase transformations, thermal expansion, or chemical reactions. Under the small strain assumption, the total strain tensor *ε* is thus decomposed into two components: the elastic strain $\varepsilon_{e}$ that contributes to macroscopic stress and the inelastic Eigenstrain $\varepsilon_{p}$ induced by precipitates—namely, the volume expansion of corrosion products. Damage reduces the material’s stress-bearing capacity, yielding the Cauchy stress tensor σ as follows [32]:

(16) $$\sigma =g(d)D_{e}:\varepsilon_{e}=g(d)D_{e}:(\varepsilon -\varepsilon_{p}),$$

In the chemical-mechanical coupling calculations of this model, the force ultimately driving concrete cracking is attributed to the volume expansion generated during reactant formation. When all iron at a given material point is converted to Fe(OH)_3_, the resulting strain is designated as $\varepsilon_{v3}$. The formula for calculating the expansion strain at this limit state is as follows:

(17) $$\varepsilon_{v3}=\frac{1}{3}\left(\frac{V_{p3}}{V_{\mathrm{Fe}}}-1\right)=\frac{1}{3}\left(\frac{\rho_{\mathrm{Fe}}M_{p3}}{\left(1-r_{0}\right)\rho_{p3}M_{\mathrm{Fe}}}-1\right),$$

$V_{\mathrm{Fe}}$ represents the molar volume of elemental iron; $\rho_{\mathrm{Fe}}$ is the density of iron; and $V_{p3}$ denotes the molar volume of the final product Fe(OH)_3_. The resulting Fe(OH)_3_ is also considered a porous medium. Here, its porosity is defined as $r_{0}$, with a value of 0.16.

The surrounding concrete matrix tightly encapsulates these newly formed corrosion products in rebar-concrete structures. This confinement induces compressive stresses at the steel-concrete interface and tensile stresses within the concrete. To precisely determine the actual stress and strain fields under these constraints, we employ Eshelby’s equivalent inclusion theory, derived from micromechanics. The actual Eigenstrain derived under this theory represents an equivalent result accounting for the mechanical constraints imposed by the surrounding matrix. Its expression is as follows [50,51]:

(18) $$\varepsilon_{p}=\frac{3(1-\nu )K_{p}}{(1+\nu )K_{p}+(2-4\nu )K}\varepsilon_{v_{3}},$$

where *K* and *Kp* are the bulk moduli of concrete and corrosion, respectively, their Young’s and Poisson ratios are given by $K_{i}\;={\;E}_{i}/(3(1-2\nu_{i}))$. Based on the volume fraction $\theta_{p}$ of the precipitated phase, the mechanical properties of the rust-filled region (E,ν) are interpolated between the mechanical properties of rust-free concrete ($E_{c}$,$\nu_{c}$) and rusted concrete ($E_{p}$,$\nu_{p}$). Using a blending rule, the effective properties are calculated as:

(19) $$E=(1-\theta_{p})E_{c}+\theta_{p}E_{p},\;\nu =(1-\theta_{p})\nu_{c}+\theta_{p}\nu_{p},$$

The overall governing equation for damage and mechanical behavior is as follows:

(20) $$\left\{\begin{array}{l} \nabla \cdot \left(g(d)D_{e}:(\nabla_{s}u-\varepsilon_{p})\right)+\bar{b}=0\;\\ \left(g(d)D_{e}:(\nabla_{s}u-\varepsilon_{p})\right)\cdot n-f=0 \end{array}\right.\begin{array}{c} in\;\Omega \\ on\;\partial \Omega_{f} \end{array},$$

(21) $$\left\{\begin{array}{l} \nabla \cdot \frac{2l_{0}}{c_{0}}G_{f}\nabla d-g^{\prime }\left(d\right)Y\left(t\right)-\frac{G_{f}}{\pi l_{0}}\alpha^{\prime }(d)\leq 0\;\\ \frac{2l_{0}}{c_{0}}G_{f}\nabla d\cdot n_{\Gamma }\geq 0 \end{array}\right.\begin{array}{c} in\;\Gamma \\ on\;\partial \Gamma \end{array},$$

Equation (20) describes the coupling relationship between mechanical equilibrium within the domain and along the boundary $\partial \Omega_{f}$ and phase-field damage. g(d) is the damage degradation function, reflecting the influence of the phase field damage variable d on material stiffness. $D_{e}$ is the fourth-order elastic stiffness tensor, $\nabla_{s}u$ is the strain tensor, and $\bar{b}$ and f are the body and boundary forces, respectively. Equation (21), based on variational phase field theory, describes the coupled evolution of phase field damage and fracture energy release within the domain Γ and along its boundary ∂Γ. The geometric constant $c_{0}\;$= π, while n and $n_{\Gamma }$ denote the unit normal vectors on the boundaries of the mechanical domain Ω and the phase field damage domain Γ, respectively.

### 2.6. Effect of Corrosion and Damage on Diffusion

The porosity (ϕ) of concrete is regarded as a dynamic state variable, its variation determined by two opposing physical mechanisms. Pore clogging: solid corrosion products generated by electrochemical reaction continuously fill the original pore network of concrete, leading to a reduction in local porosity. Damage-induced porosity: when corrosion products’ expansion stress exceeds the matrix’s tensile strength, micro-cracks form and propagate. The volume of these newly formed cracks manifests macroscopically as an increase in porosity. The formula for calculating Porosity changes due to corrosion products is as follows:

(22) $$\varphi_{p3}=\frac{M_{p3}C_{p3}}{\rho_{p3}(1-r_{0})},$$

$C_{p3}$, $M_{p3}$, and $\rho_{p3}$ represent the molar concentration, molar mass, and density of Fe(OH)_3_, respectively, while r_0_ denotes the porosity of Fe(OH)_3_.

This study, utilizing a phase-field damage theory and a logistic function, constructed a porosity evolution model—with parameters like the damage threshold ($d_{0}$ = 0.59) and steepness parameter (*k* = 13.04) calibrated against experimental data —to accurately replicate the nonlinear physical mechanism where porosity gradually increases before accelerating sharply upon surpassing a critical damage threshold in concrete.

(23) $$\varphi_{L}^{*}=\varphi_{L,0}+\frac{1-\varphi_{L,0}}{1+\exp\left(-k(d-d_{0})\right)}\cdot d^{n}-\varphi_{p3},$$

## 3. Numerical Modeling Corrosion-Induced Degradation in Unsaturated Reinforced Concrete Structure

### 3.1. Numerical Model and Parameters

**Figure 4:**
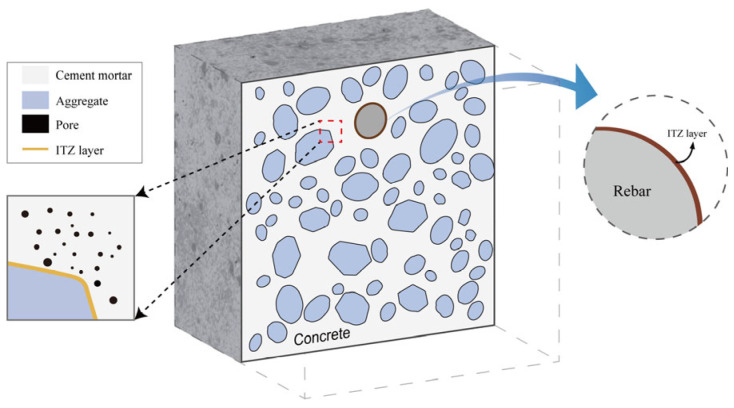
Numerical model.

Figure 4 depicts the numerical model including polyhedral aggregates and rebar. The model delineated in this study is calibrated and validated utilizing experimental data from Ye et al. [52]. In the initial experiment, the rebar was subjected to corrosion via a dry–wet cycle, followed by placement of the specimens in a test chamber with 80% relative humidity and a temperature of 33 °C to expedite the corrosion process. The model’s dimensions are 100 by 100 mm, featuring a rebar diameter of 10 mm. The concrete cover thicknesses on the top and right side are 10 mm and 25 mm, respectively. The inter-tooth zone (ITZ) thickness between the aggregate and matrix is 0.3 mm, whereas the ITZ thickness between the rebar and matrix is 0.2 mm [32].

The model’s base is subjected to complete restrictions in both horizontal and vertical directions (i.e., fixed constraints). To replicate the 32-day wet-dry cycle during the corrosion initiation phase, periodic moisture and temperature conditions are imposed on the border exposed to the external environment. A piecewise function establishes the moisture saturation and temperature boundaries at 0.9 and 25 °C, respectively, during the wet phase, and at 0.2 and 50 °C during the dry period. The concept posits that chloride ions and oxygen can effortlessly traverse all exterior limits for chemical transport. The boundary concentration of chloride ion was calculated to be 37.85 g/L based on the 6% mass fraction NaCl solution utilized in the experiment. The oxygen content at the concrete surface was established at the equilibrium level under standard air circumstances, specifically 0.268 mol/m^3^. In the corrosion propagation phase, specimens were placed in an environmental chamber for expedited corrosion at 33 °C and 80% relative humidity. At this stage, boundary and internal saturation levels were upheld as constant values throughout the model.

The initial material properties allocated to these phases are essential for the simulation. The initial porosity (phi) of the cement mortar matrix is not a mere constant; it is influenced by the water–cement ratio (*w*/*c*) and the initial degree of hydration α_0_ = 0.75, as delineated in Equation (4). The principal factors regulating unsaturated hygro-thermal transport, including the reference moisture diffusion coefficients for drying $D_{w0}^{d}$ and wetting $D_{w0}^{d}$, are delineated in Table 2. The unique mechanical parameters of the aggregate, mortar, and interfacial transition zone (ITZ) phases—comprising Young’s modulus, Poisson’s ratio, tensile strength, and fracture energy—are presented in Table 3.

The multi-ion reactive transport module depends on the initial concentrations and diffusion coefficients of the nine chemical species (O_2_, Cl^−^, Na^+^, etc.), with particular values for the mortar and interfacial transition zone phases detailed in Table 1. The fundamental electrochemical parameters influencing corrosion kinetics, including the Tafel slopes $\beta_{a}^{\mathrm{Fe}}$, $\beta_{c}^{\mathrm{ORR}}$ and the exchange current densities $i_{0}^{\mathrm{Fe}}$, $i_{0}^{\mathrm{ORR}}$, are presented in Table 4.

### 3.2. Model Calibration and Validation

**Figure 5:**
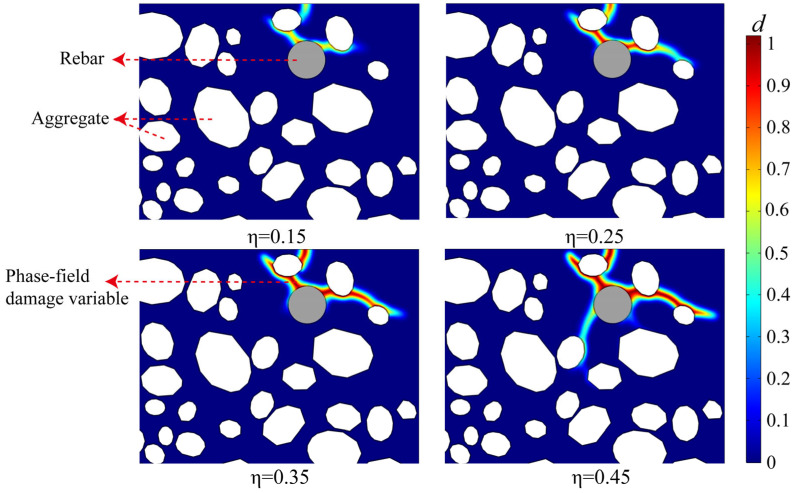
The evolution of the crack network shown by the phase-field damage variable (d) at different rebar corrosion degrees (η = 0.15, 0.25, 0.35, and 0.45).

To guarantee the dependability and predicted precision of the proposed multiphysics framework, the model was rigorously calibrated against the experimental data presented by Ye et al. [52]. The calibration method concentrated on the essential parameters that dictate damage evolution in the phase-field model, which are not readily ascertainable from direct material testing. The damage threshold (d_0_ = 0.59) and the steepness parameter (k = 13.04), which regulate the onset and propagation of micro-cracks (refer to Equation (23)), were modified iteratively. The model was deemed effectively calibrated when the numerically projected fracture initiation time and the ensuing crack propagation path (shown in Figure 5) exhibited a strong correlation with the experimental results recorded in [52]. All other material parameters, encompassing hygro-thermal transport (Table 2), mechanical properties (Table 3), multi-ion transport (Table 1), and electrochemical kinetics (Table 4), were directly sourced from the existing literature or the reference experiment [52]. These characteristics were excluded as calibration variables, hence guaranteeing the model’s physical realism and robustness.

## 4. Results and Discussion

### 4.1. Result of Damage Evolution and Crack Propagation Path

Figure 5 illustrates the comprehensive progression of crack initiation, propagation, and the subsequent development of a complex network within the concrete cover when the corrosion rate of the rebar (η) escalates, employing a contour plot of the phase field damage variable (d). This outcome indicates that the behavior of fracture mechanics is governed by non-uniform corrosion and is profoundly affected by the microstructure of the concrete. In the preliminary corrosion phase (η = 0.25), deterioration initially arises in the most susceptible interfacial transition zone (ITZ). The minimal buildup of corrosion products creates expansion stresses that are adequate only to cause slight damage in areas of significant stress concentration, without resulting in continuous fissures. As the corrosion rate escalates to η = 0.25, the affected region enlarges considerably. The earliest microdamage regions commence propagation along trajectories of maximal energy release, resulting in the formation of a primary fracture.

Crack propagation reached a mature phase as the corrosion rate advanced to η = 0.35 and η = 0.45. The principal fissure expanded and elongated, subsequently diverging to create a more intricate network of minor fissures. At η = 0.45, many cracks are evident extending outward from the principal corrosion source (the upper section of the rebar) and engaging with neighboring aggregate interfaces. These fissures may eventually permeate the concrete surface, resulting in macroscopically discernible cracking. This procedure illustrates the entire failure sequence: from the buildup of micro-damage, through the erratic advancement of primary fractures, to the ultimate development of a widespread crack network.

### 4.2. Discussion

**Figure 6:**
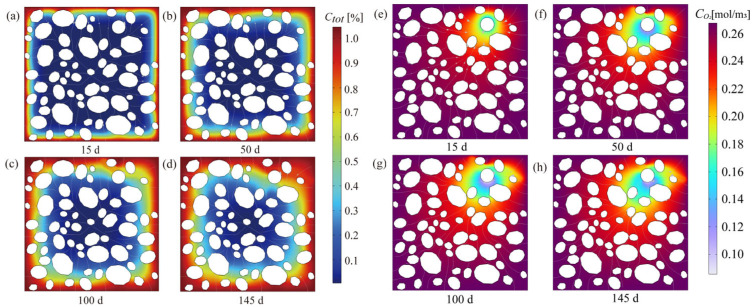
Spatiotemporal evolution of key concentration fields. (**a**–**d**) Total chloride content (Ctot) at 15 d, 50 d, 100 d, and 145 d; (**e**–**h**) Oxygen concentration (CO_2_) at 15 d, 50 d, 100 d, and 145 d.

As initial micro-cracks form (e.g., at η = 0.25), they do not merely represent mechanical failure; they function as new, high-velocity pathways (relative to the undamaged mortar matrix) for corrosive species. Figure 6 systematically illustrates the spatiotemporal evolution of total chloride content (*Ctot*) and oxygen concentration (*C*_*O*_2__) within the mesoscale representative volume element (RVE) from 15 days to 145 days. In the early corrosion phase (15d to 50d), chloride ions primarily penetrate the cement mortar matrix through concentration gradients, resulting in a distinct diffusion front moving inward (Figure 6a,b). Concurrently, oxygen is consumed at the cathode regions, evidenced by the localized low concentration “sinks” around the rebar at 15d and 50d (Figure 6e,f). These regions of minimum C_O2_ correspond to the most active cathodic reaction sites.

As corrosion advances and products accumulate, the resulting microcrack network becomes apparent in the later stages (100d to 145d). These cracks critically alter the transport properties of the concrete. As shown in Figure 6c,d, the fissures act as preferential transport conduits (or “fast lanes”) for chloride ions, significantly accelerating their ingress rate into the core zone and establishing conditions for persistent corrosion.

Furthermore, the macro-cracks markedly improve the efficiency of gas-phase oxygen transport from the external environment. This enhanced supply mitigates the reaction inhibition caused by rapid oxygen consumption at the cathode, thereby preserving a comparatively higher steady-state local *C*_*O*_2__ at the rebar surface (Figure 6g,h) despite vigorous cathodic activity. This observation quantitatively confirms the damage-induced positive feedback loop: microcracking expedites the supply of corrosive agents (Cl^−^ and O_2_), leading to an accelerated electrochemical reaction rate and subsequent nonlinear structural degradation.

Table 5 offers a comprehensive comparison with contemporary state-of-the-art phase-field corrosion models to further contextualize the contribution of this work. As demonstrated, despite substantial advancements in integrating electrochemical and mechanical domains [33], a prevalent constraint is the assumption of completely saturated concrete. The framework established by Korec et al. [31] effectively approximated mesoscale heterogeneity; nevertheless, it failed to incorporate dynamic hygro-thermal transport and the unsaturated conditions commonly found in actual structures.

## 5. Conclusions

From the conducted numerical simulations and studies, the following principal conclusions can be derived:

1. Scientific Findings in Materials Science: The research effectively created and confirmed a thorough Hygro-Thermo-Electro-Chemo-Mechanical (HTECM) phase-field model. This model precisely elucidates the essential degradation mechanisms, highlighting the pivotal function of the damage-induced “transport-corrosion” positive feedback loop, wherein micro-cracking significantly accelerates local moisture and ion transport, resulting in a nonlinear escalation of deterioration.
2. Engineering Applications and Resource Requirements: The model offers a precise numerical tool for actual engineering applications.

For the assessment of “residual service life”: The platform enables engineers to advance beyond basic empirical models by offering quantitative forecasts of crack initiation time and the ensuing progression of crack width and corrosion depth under defined, dynamic environmental conditions. This is crucial for accurate service life prediction and the planning of maintenance activities.

In “concrete design,” the model operates as a resilient “virtual laboratory.” It enables designers to proactively evaluate and compare the long-term durability of various design options—such as altering the concrete cover thickness, adjusting the concrete mix design (e.g., water-to-cement ratio, which influences porosity), or assessing the effectiveness of corrosion inhibitors—prior to construction.

Necessary resources: The suggested platform, being a fully linked, multi-physics finite element model, necessitates substantial computational resources and knowledge. It depends on specialist numerical software that can resolve the intricate system of partial differential equations, unlike simpler, less precise empirical formulae.

3. Future Development Prospects: This study provides a solid basis for subsequent research. The existing framework must be augmented to include additional long-term effects, such as concrete creep, bond–slip between rebar and concrete, and the interaction of freeze–thaw cycles, to improve its forecast precision for actual constructions.

## Figures and Tables

**Table 1:** Diffusion coefficient and concentration in the model [33,34,44].

Parameters	O_2_	Cl^−^	Na^+^	K^+^	OH^−^	Fe^2+^
Initial concentration (mol/m^3^)	0.268	0	9.53	6.56	16.35	0
Diffusion coefficient in mortar (×10^−11^ m^2^/s)	4.5 × 10^3^	2.03	1.79	2.63	7.08	0.965
Diffusion coefficient in ITZ (×10^−11^ m^2^/s)	3.6 × 10^4^	13.65	8.95	13.15	35.4	4.825

**Table 2:** Parameters for Concrete Moisture Diffusion Coefficient [37,38,45].

Parameter	Value
Moisture reference diffusion coefficient during drying process, $D_{w0}^{d}$ (m^2^/s)	8.31 × 10^−10^
Moisture reference diffusion coefficient during wetting process, $D_{w0}^{s}$ (m^2^/s)	4.05 × 10^−11^
Volume fraction of aggregates, $v_{\mathrm{agg}}$	Case-dependent
The ratio of minimum $D_{w}^{c}$ to $D_{w0}^{d}$, $\gamma_{0}$	0.025
the relative humidity when $D_{w}^{c}\;$=${\;D}_{w0}^{d}$/2, $s_{c}$	0.8
parameter characterizing the drop and rise spread during the drying process $n_{1}$	6
parameter characterizing the drop and rise spread during the wetting process $n_{2}$	6
water–cement ratio w/c	Case-dependent
the initial degree of hydration α_0_	0.75

**Table 3:** Calculation parameters of different phases in concrete [33,53].

Phase	Young’s Modulus (GPa)	Poisson’s Ratio	Tensile Strength (MPa)	Fracture Energy (N/m)
Aggregate	80.0	0.2	-	-
Mortar	30.0	0.167	30	40
ITZ	15.0	0.167	15	20

**Table 4:** Electrochemical parameters [33,54,55].

Parameters	Value	Unit
Anodic Tafel slope, $\beta_{a}^{\mathrm{Fe}}$	0.09	V/dec
Anodic equilibrium potential, $E_{\mathrm{eq}}^{\mathrm{Fe}}$	−0.78	V
Anodic exchange current density, $i_{0}^{\mathrm{Fe}}$	3 × 10^−4^	A/m^2^
Cathodic Tafel slope, $\beta_{c}^{\mathrm{ORR}}$	−0.14	V/dec
Cathodic equilibrium potential, $E_{\mathrm{eq}}^{O_{2}}$	1 × 10^−5^	V
Cathodic exchange current density, $i_{0}^{\mathrm{ORR}}$	2.5	A/m^2^

**Table 5:** Comprehensive comparison of phase-field corrosion models.

Feature	This Study (HTECM Model)	Korec et al. [31]	Qiu et al. [33]
Physics Coupling	H-T-E-C-M (Fully coupled)	Chemo-Mechanical	Electro-Chemo-Mechanical
Saturation State	Unsaturated (Dynamic S(t))	Saturated	Saturated
Environmental Driver	Dynamic Temp. & Humidity, Constant Chloride Flux	Constant Chloride Flux	Constant Chloride Flux
Material Heterogeneity	Mesoscale (Polygonal Aggregates)	Homogeneous (Macroscale)	Mesoscale (Circular Aggregates)

## Data Availability

The original contributions presented in this study are included in the article. Further inquiries can be directed to the corresponding author.

## Abbreviations

The following abbreviations are used in this manuscript:

RC	Reinforced Concrete
PFM	Phase-Field Method
HTECM	Hygro-Thermo-Electro-Chemo-Mechanical
ITZ	Interfacial Transition Zone
PF-CZM	Phase-Field Regularized Cohesive Zone Model
FPZ	Fracture Process Zone
*w*/*c*	Water–Cement Ratio
